# Spongy Bougies

**Published:** 1854-12

**Authors:** 


Spongy Bougies.—M. Alquie has constructed bougies of the following kind, viz: Round a whalebone bougid is rolled a sponge; this is reduced by compression to a third of its volume; it is then covered with gold beaters' skin, and is covered with the salve used in ordinary bougies. The instrument when about to be used is dipped in water, and then, when its point has been touched with cerate, is introduced into the urethra. The sponge slowly imbibes the water, and swells so as to distend the urethra, supposing this to be the canal operated upon. Whether there is then any difficulty in withdrawing the instrument, or any danger of injuring the urethra in so doing, does not appear.

Ricord has used this instrument in two cases of stricture; it was introduced easily, but could not be borne more than an hour; it was withdrawn without difficulty, as the swelling of the sponge was not great.

M. Alquie points out various other possible applications of the sponge-bougies. He alludes to a case in which the fractured nasal bones were held in place easily by its means.—*Ibid.*

